# Biparental Resequencing Coupled With SNP Genotyping of a Segregating Population Offers Insights Into the Landscape of Recombination and Fixed Genomic Regions in Elite Soybean

**DOI:** 10.1534/g3.113.009589

**Published:** 2014-01-29

**Authors:** Ying-hui Li, Yu-lin Liu, Jochen C. Reif, Zhang-xiong Liu, Bo Liu, Michael F. Mette, Ru-zhen Chang, Li-juan Qiu

**Affiliations:** * The National Key Facility for Crop Gene Resources and Genetic Improvement (NFCRI)/Key Lab of Germplasm Utilization (MOA), Institute of Crop Science, Chinese Academy of Agricultural Sciences, 100081 Beijing, China; † Department of Cytogenetics and Genome Analysis, Leibniz Institute of Plant Genetics and Crop Plant Research (IPK), Corrensstrasse 3, D-06466 Stadt Seeland, Gatersleben, Germany

**Keywords:** soybean, biparental segregating population, resequencing, SNP, linkage map

## Abstract

Identification of genes underlying agronomic traits is dependent on the segregation of quantitative trait loci (QTL). A popular hypothesis is that elite lines are becoming increasingly similar to each other, resulting in large genomic regions with fixed genes. Here, we resequenced two parental modern elite soybean lines [*ZhongHuang13* (ZH) and *ZhongPin03-5373* (ZP)] and discovered 794,876 SNPs with reference to the published Williams82 genome. SNPs were distributed unevenly across the chromosomes, with 87.1% of SNPs clustering in 4.9% of the soybean reference genome. Most of the regions with a high density of SNP polymorphisms were located in the chromosome arms. Moreover, seven large regions that were highly similar between parental lines were identified. A GoldenGate SNP genotyping array was designed using 384 SNPs and the 254 recombinant inbred lines (F_8_) derived from the cross of ZP × ZH were genotyped. We constructed a genetic linkage map using a total of 485 molecular markers, including 313 SNPs from the array, 167 simple sequence repeats (SSRs), 4 expressed sequence tag–derived SSRs, and 1 insertion/deletion marker. The total length of the genetic map was 2594.34 cM, with an average marker spacing of 5.58 cM. Comparing physical and genetic distances, we found 20 hotspot and 14 coldspot regions of recombination. Our results suggest that the technology of resequencing of parental lines coupled with high-throughput SNP genotyping could efficiently bridge the genotyping gap and provide deep insights into the landscape of recombination and fixed genomic regions in biparental segregating populations of soybean with implications for fine mapping of QTL.

Theoretical considerations suggest that an important prerequisite for a high power of quantitative trait loci (QTL) detection within biparental crosses is that the genetic distance between adjacent polymorphic markers is less than 10 cM (Piepho 2000). Moreover, it is often important to saturate the genetic linkage map in genomic regions where QTL are located to develop stable diagnostic markers for marker-assisted selection. Despite the accessibility of established molecular markers in major crops such as soybean, it is often difficult to find a sufficiently dense set of polymorphic markers for a specific biparental cross. The current approaches to screen available established molecular markers for polymorphism are time-intensive and labor-intensive, and there might be genomic regions that are barely polymorphic between parental materials, which are particularly probable among related elite lines.

Many molecular markers are available for soybean [*Glycine max* (L.) Merr.] and have been used to establish integrated genetic linkage maps. The first and second versions of the soybean integrated genetic linkage map were based on isozyme markers, restriction fragment length polymorphism, random amplified polymorphic DNA, amplified fragment length polymorphisms, and simple sequence repeats (SSR) (Cregan *et al. et al.* 1999; Song *et al.* 2004). More recently, several thousand single-nucleotide polymorphisms (SNPs) markers were added to the integrated genetic map (Choi *et al.* 2007; Hyten *et al.* 2010), bringing the total of integrated markers to 3641. Focusing on specific traits, a series of biparental segregating populations have been developed for QTL mapping, mostly derived from crosses between two elite cultivars. The use of crosses among elite cultivars, however, is potentially afflicted with a high proportion of fixed QTL. Studies based on pedigree data suggested that most released cultivars possess high genetic similarity because of use of only a few prominent founder lines in soybean breeding programs (Gizlice *et al.* 1994; Xiong *et al.* 2007). This genetic similarity is particularly pronounced for cultivars originating from the same geographical region.

Integrating next-generation sequencing technology with the development of resequencing analysis provides an opportunity to quickly identify large numbers of population-specific SNPs (Huang *et al.* 2010). For instance, 150 rice recombinant inbred lines were resequenced and a SNP-based ultrahigh-density linkage map was constructed (Huang *et al.* 2009). Using genotyping by sequencing technology, 30,984 SNPs were added to a rice recombinant inbred lines (RIL) population for QTL mapping (Spindel *et al.* 2013), suggesting that a sequencing-based approach is fast and economic in terms of data collection. Moreover, genotyping by sequencing facilitates increased precision to determine recombination breakpoint compared to previously constructed genetic linkages maps based on sparse PCR-based markers (Huang *et al.* 2009; Spindel *et al.* 2013).

Soon after the publication of a soybean reference genome sequence (Schmutz *et al.* 2010), resequencing developed into a major technology for genome-wide polymorphism discovery. Until now, 32 cultivated soybean [*Glycine max* (L.) Merr.] accessions, including two parental lines [*ZhongHuang13* (ZH) and *ZhongPin03-5373* (ZP)] used in this study, have been resequenced, and several millions of SNPs have been identified (Lam *et al.* 2010; Li *et al.* 2013). In this study, we developed a customized SNP array based on the resequencing data from ZH and ZP and applied it to genotype 254 F_8_ plants from a segregating biparental soybean population using a high-throughput Illumina GoldenGate genotyping platform (Hyten *et al.* 2008). Combining information from the genetic linkage map and the resequencing data on the genetic divergence among the two parents, we constructed a soybean linkage map and observed the presence of recombination hotspot and coldspot regions and of several large genomic regions that were genetically fixed.

## Materials and Methods

### Mapping population

A cross was made between the modern cultivar ZH (provided by Professor Lianzheng Wang, Institute of Crop Science, Chinese Academy of Agricultural Sciences ICS-CAAS, Beijing, China) as the male parent and elite line ZP (developed by ICS-CAAS) as the female parent. ZH is widely planted in China for elite agronomic traits such as high grain yield, large seed weight, low plant height, and wide adaptability. ZP is a line with resistance to soybean cyst nematode race 4 (Wei *et al.* 2011). A population consisting of 254 recombinant inbred lines at F_8_ generation was developed from the cross of ZP × ZH. Genomic DNA was extracted from young leaves of 10 F_8_ seedlings per inbred line using a DNAquick Plant System (Tiangen Biotech Beijing, Co., Ltd.). For resequencing analysis, total genomic DNA of ZH and ZP was extracted from fresh leaves of dark-grown plants at the first trifoliolate stage using a DNeasy Plant Mini Kit (QIAGEN).

### Whole-genome sequencing, alignment, and SNP calling

To screen for SNPs between parents, ZH and ZP were resequenced by BGI-Shenzhen (Shenzhen, China) using the Illumina Solexa system. DNA library construction, short reads derivation, alignment onto the soybean reference genome (*G. max* var. Williams 82; www.phytozome.net) (Schmutz *et al.* 2010), and SNP calling had been described previously (Li *et al.* 2013). The repeat mapped, unique mapped, and unmapped short reads were identified by aligning these reads onto the reference genome using SOAP2 (Li *et al.* 2009) with the parameters reported previously (Li *et al.* 2013). Only unique mapped sequences were used to call SNPs. The type and location of SNPs with regard to the reference sequence were determined based on the Bayesian theory and the maximum likelihood estimation method. The certain criteria for improving the accuracy of each SNP were also followed during the previous study (Li *et al.* 2013). Based on the open reading frame in the gene prediction, we used a self-developed Perl script to identify the synonymous and nonsynonymous site after we replaced the individual genotyping at each SNP site. All sequence reads and the SNPs were included in the Sequence Read Archive under accession number SRP015830 and the Database of Short Genetic Variations (dbSNP) with batch ID 1058942, respectively (Li *et al.* 2013).

### Marker selection and polymorphism detection

#### SSR, EST-SSR, and insertion/deletion markers:

In total, 928 molecular markers, including 880 simple sequence repeats (SSRs), 37 EST-SSRs, and 1 insertion/deletion (InDel) marker, randomly distributed over the 20 genetic linkage groups of soybean were screened for polymorphisms between the parental elite inbred lines ZH and ZP. Among 880 SSRs, 789 SSRs were from the integrated linkage map of soybean (Song *et al.* 2004) and 101 were newly developed markers (Song *et al.* 2010). All the SSR primer sequences were obtained from the soybean database Soybase (http://www.soybase.org/). In addition, 37 EST-SSR and 1 InDel marker, rhg1-I4, were included in the analysis (Liu *et al.* 2010; Nan *et al.* 2010). From these markers, a total of 179 were robustly polymorphic between ZH and ZP and used to genotype the complete set of 254 recombinant inbred lines.

Polymerase chain reactions (PCRs) were conducted on a Model MG96^+^ thermal cycler from LongGene Scientific Instruments, Co. Ltd. (Hangzhou, China). Each reaction volume of 20 μl consisted of 50–100 ng genomic DNA template, 2 μl 10×PCR buffer (containing Mg^2+^), 1.5 μl 2 mM of each dNTP, 1.5 μl 2 mM of forward and reverse primers, and 1 U *Taq* polymerase. The program for PCR was as follows: 5 min at 95° followed by 35 cycles of 30 sec at 94°; 30 sec at a primer-specific optimized annealing temperature; 30 sec at 72°; and a final extension at 72° for 8 min. The PCR products were separated on 6% denaturing polyacrylamide gels and visualized by silver staining.

#### SNPs:

The primary linkage genetic map constructed using 179 polymorphic SSRs, EST-SSRs, and InDel markers included 45 large gaps with distances more than 20 cM, which partitioned 20 normal soybean linkage groups into 45 linkage groups. To bridge these gaps, SNP markers were selected from the genomic region of these gaps. Of 384 SNPs selected, most (240; 62.5%) were nonsynonymous or nonsense. In addition, all of 384 SNPs were with a design ability rank score >0.6 and a pre-evaluation of 60 bp of upstream and downstream flanking regions. Then, an Illumina GoldenGate SNP array (Illumina, San Diego, CA; www.illumina.com) was developed using these SNPs. The SNP array was used to genotype the complete set of 254 recombinant inbred lines and the two parental lines using the Illumina GoldenGate assay system following the manufacturer’s protocol. For each SNP, the lowest acceptable scores were set to 80% for GenCall and to 0.6 for GenTrain to separate homozygote and heterozygote clusters.

### Genetic linkage map construction

A genetic linkage map was constructed using the software package IciMapping V3.1 (Li *et al.* 2008) (freely available from www.isbreeding.net). For grouping, the threshold for limit of detection (LOD) scores was set to 3.0 and the threshold for marker distance was defined as 50 cM. The recombination frequency was converted to genetic map distances (in cM) using the Kosambi mapping function (Kosambi 1943). SERiation (SER) (Buetow and Chakravarti 1987) was used as the ordering algorithm. The criterion of the sum of adjacent LOD scores was used for rippling after ordering each marker sequence. Linkage groups were assigned to chromosomes based on the publicly available genetic linkage map (Song *et al.* 2004) and the soybean genome reference sequence (Schmutz *et al.* 2010). The genetic linkage map was graphically displayed using MapChart2.2 (Voorrips 2002). We used previously identified pericentromeric regions and chromosome arms (Du *et al.* 2012).

## Results

### SNPs discovery

**Figure 1:**
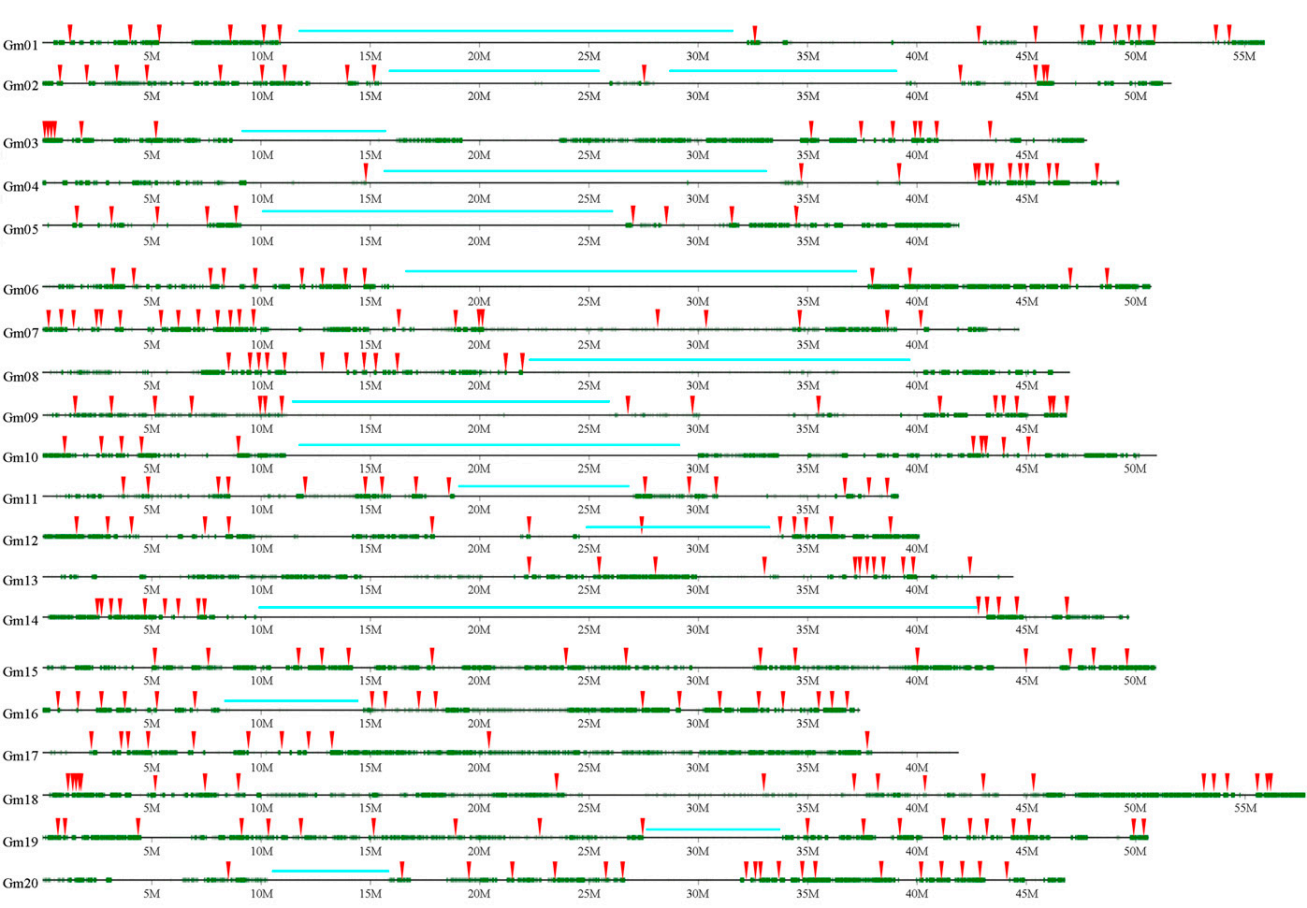
Distribution of SNPs discovered by resequencing between parental inbred lines ZH and ZP across 20 soybean reference chromosomes on the physical map. SNPs are displayed with green lines. The 313 SNPs used to construct a genetic linkage map are indicated by red arrows. Extended identical regions that are mostly void of SNPs are marked by blue bars.

**Figure 2:**
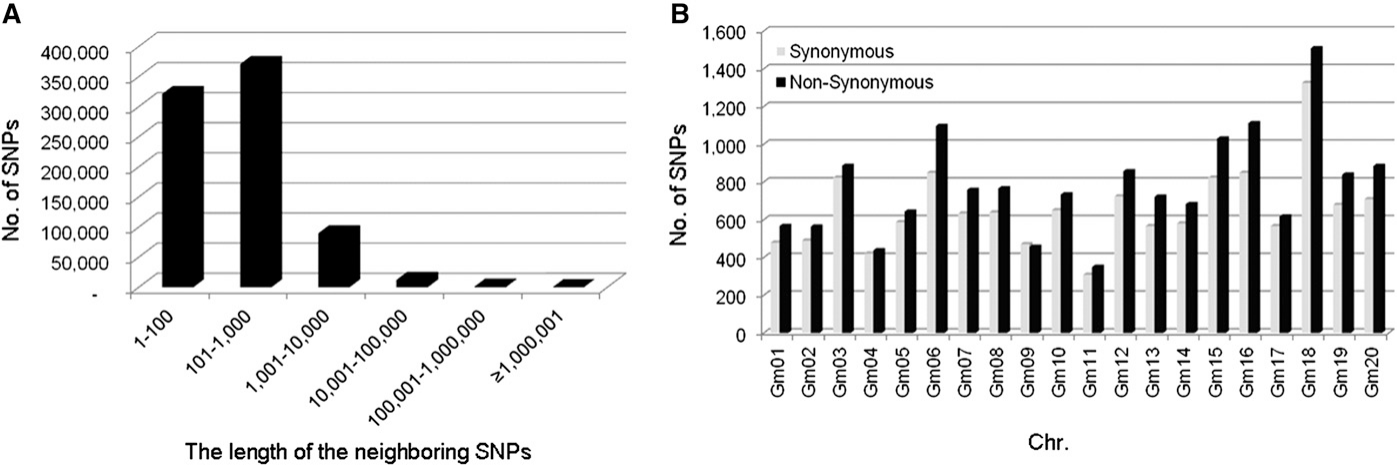
Distribution of the length between adjacent SNPs (A) and the frequency of synonymous and nonsynonymous SNPs on the chromosomes (B).

A total of 928 established molecular markers, including 890 SSRs, 37 EST-SSRs, and 1 InDel, were first screened for polymorphisms between the parents ZH and ZP. Only 179 (19.3% of total markers tested) showed stable amplification and clear polymorphic bands, including 173 SSRs, 5 EST-SSRs, and the InDel marker. When a soybean linkage map was constructed using these sparse markers, the 20 normal soybean linkage groups were broke into 45 linkage groups (data not shown) because of 25 large gaps with distances more than 50 cM. To increase the number of available polymorphic markers for bridging the gaps in the genetic map for the biparental cross of interest, the resequenced genomes of the two parental modern lines ZP and ZH were analyzed. A total of 2980 Mb and 5240 Mb raw sequence data consisting of sequence reads of 75 bp in length were obtained from ZP and ZH, respectively. After filtering out repetitive as well as unmapped sequences, the remaining 2700 Mb and 4470 Mb of low copy genomic sequences, respectively, were aligned to the soybean reference genome (variety Williams 82) pseudomolecules (www.phytozome.net) (Schmutz *et al.* 2010) for SNP discovery. The sequence data from ZP represented 87.5% of the reference genome with 2.82-fold coverage, whereas the sequence data from ZH represented 93.4% of the reference genome with 4.68-fold coverage. The ZP sequences exhibited a lower average rate (0.66%) of mismatches with the reference sequences than the ZH sequences (0.88%). After sequence alignment and SNP determination with regard to the reference sequence, 1,127,624 SNPs were identified discriminating between the genomes of ZP or ZH and the reference. Of them, 70.5% (794,876 SNPs) were detected between ZP and ZH with regard to the reference sequence, with an average of 39,744 SNPs in each chromosome (Table 1; http://www.ncbi.nlm.nih.gov/SNP/snp_viewTable.cgi?handle= NFCRI_ MOA_CAAS), with a distribution that differed significantly among the chromosomes. The highest number of SNPs (67,921) was on Gm18 and the lowest number (14,682) was on Gm04. Moreover, SNPs were unevenly distributed within chromosomes (Figure 1); 87.1% (692,085) of all SNPs had a distance of less than 1000 bp to its nearest neighboring SNP (Figure 2A). The 4.9% of the soybean reference genome containing multiple adjacent SNPs between the two parents were mostly located on chromosome arms.

**Table 1:** Distribution pattern of 794,876 SNPs discovered by resequencing of ZP and ZH

Chromosome	Intergenic Region	Genic Region						Total
		CDS			Intron	UTR	Subtotal	
		Subtotal	Synonymous	Nonsynonymous				
Gm01	28,251	1044	478	566	2678	513	4235	32,486
Gm02	19,147	1052	489	563	3380	614	5046	24,193
Gm03	46,507	1707	822	885	5463	913	8083	54,590
Gm04	14,682	857	420	437	2638	549	4044	18,726
Gm05	19,767	1228	586	642	3726	762	5716	25,483
Gm06	40,328	1944	848	1096	5586	1045	8575	48,903
Gm07	30,310	1389	632	757	3978	800	6167	36,477
Gm08	21,266	1403	638	765	4033	786	6222	27,488
Gm09	15,652	926	470	456	2721	561	4208	19,860
Gm10	29,261	1383	650	733	4038	797	6218	35,479
Gm11	18,043	657	308	349	2138	404	3199	21,242
Gm12	29,900	1579	723	856	4592	874	7045	36,945
Gm13	32,622	1287	566	721	4144	896	6327	38,949
Gm14	20,283	1262	580	682	3311	645	5218	25,501
Gm15	66,834	1851	822	1029	5186	973	8010	74,844
Gm16	38,597	1959	848	1111	4606	962	7527	46,124
Gm17	41,986	1182	566	616	3551	617	5350	47,336
Gm18	67,921	2832	1324	1508	7447	1221	11,500	79,421
Gm19	47,212	1516	678	838	4493	849	6858	54,070
Gm20	39,397	1592	708	884	4935	835	7362	46,759
Total	667,966	28,650	13,156	15,494	82,644	15,616	126,910	794,876

With regard to functional annotation, most of the identified SNPs (84%) were located in intergenic regions. Among the 126,910 SNPs in genic regions, only 22.9% (28,650) were identified in coding sequences (CDS), including 13,156 synonymous SNPs and 15,494 nonsynonymous SNPs. With the exception of Gm09, the number of nonsynonymous SNPs was slightly higher than that of synonymous SNPs (Figure 2B) on 19 of the 20 chromosomes. In Gm09, the number of synonymous SNPs (470) was slightly higher than that of nonsynonymous SNPs (456).

### Genetic linkage map construction

We selected 384 SNPs and designed an array using the Illumina GoldenGate assay system to fingerprint the segregating biparental population. From the 384 SNPs, 71 were excluded from analysis because of low data quality. The remaining 313 high-quality SNPs, including 195 nonsynonymous or nonsense, 44 synonymous, and 74 intergenic SNPs, were complemented by 167 SSRs, 4 EST-SSRs, and 1 InDel from the initial set mentioned above to construct a genetic linkage map (Supporting Information, Table S1). Of the 485 markers, a total of 464 could be mapped on the soybean reference genome (Glyma1.01) covering 880 Mb (92.6%) of the genome length. The average distance between two adjacent marker loci was 1.86 Mb.

**Figure 3:**
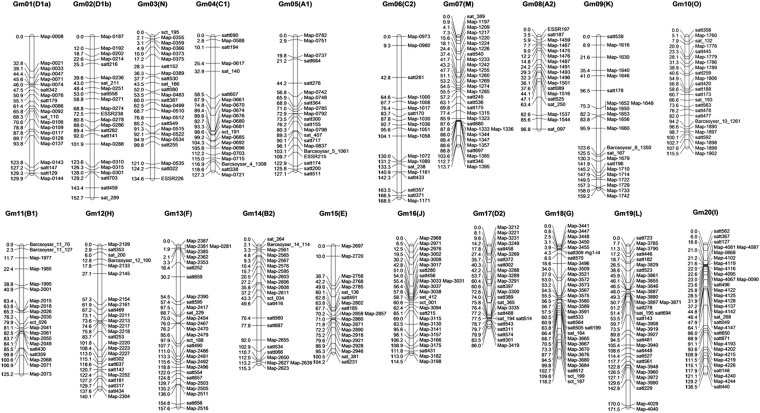
Soybean genetic linkage map constructed using 485 molecular markers.

The resulting genetic linkage map with a total length of 2594.34 cM had an average marker spacing of 5.58 cM (Figure 3). All marker loci were assigned to one of the 20 soybean linkage groups by aligning the markers to the soybean reference genome sequence (Glyma1.01) (Schmutz *et al.* 2010) and the integrated soybean genetic map (Consensus Map 4.0) (Hyten *et al.* 2010). The number of markers on Gm19 was highest with 33, followed by Gm18 and Gm 20 with 32. The lowest number of markers was detected for Gm08 with only 18 markers. The linear order of the 464 markers with information on their genomic locations was, in most cases, consistent with their order on the soybean reference genome, except for 59 markers showing deviations. Moreover, two SNP markers, Map-0090 on Gm01 and Map-0668 on Gm04, were assigned to Gm20 in the presented genetic map, cosegregating with Map-4067, Map-4081, and Map-4087, respectively. An additional marker, Map-0281 on Gm02, was assigned to Gm13.

A total of 18 gaps of >20 cM in length were present in 14 of the linkage groups, with exceptions Gm07(M), Gm08(A2), Gm10(O), Gm16(J), Gm17(D2), and Gm18(G). Most of these gaps (83.3%) were ≤30 cM in length. The largest gap was located on Gm06(C2) at 33.45 cM in length. The markers in all of the 18 gaps were sparse. A total of 48,411 SNPs were detected within the length of 127.51 Mb of 18 gaps, with a frequency of 1 SNP every 2.6-kp genomic region. This frequency is much lower than that of the other SNPs (1 SNP every 1.1-kb genomic region). Segregation distortion of each locus was estimated by the goodness of fit test, and 21 marker loci showed significant (*P* < 0.01) segregation distortions. These loci were located on Gm07(M), Gm09(K), Gm10(O), Gm18(G), and Gm20(I). The distorted segregation markers were unevenly distributed in the genome. For example, two hotspots of regions with significant segregation distortion were detected on Gm18(G) and Gm10(O) and included eight markers, from Map-3441 to satt570, and four markers, from Map-1806 to satt173, respectively.

### Landscape of fixed genomic regions and of recombinations

The rate of potential miscallings of SNPs by Sanger sequencing was determined to amount to 2.7% in soybean (Li *et al.* 2013). We examined the regions with low degrees of polymorphism identified in our study in more detail using this rate as an estimate of miscalling as threshold. Based on this criterion, 16 extended regions (>5 Mb) were scored as identical between the two parental lines, which were according to the physical map located mainly in pericentromeric regions (Figure 1). Within these regions, no SNPs were identified in seven large regions (≥1 Mb), including nucleotide positions 20,375,352–21,461,521 (1.09 Mb) on Gm01, 20,570,338–21,883,869 (1.31 Mb) and 26,841,519–27,919,452 (1.08 Mb) on Gm04, 23,354,052–24,387,140 (1.03 Mb), 30,670,807–31,966,253 (1.30 Mb), and 32,290,845–33,542,372 (1.25 Mb) on Gm06, and 21,025,993–22,030,212 (1.00 Mb) on Gm14.

**Figure 4:**
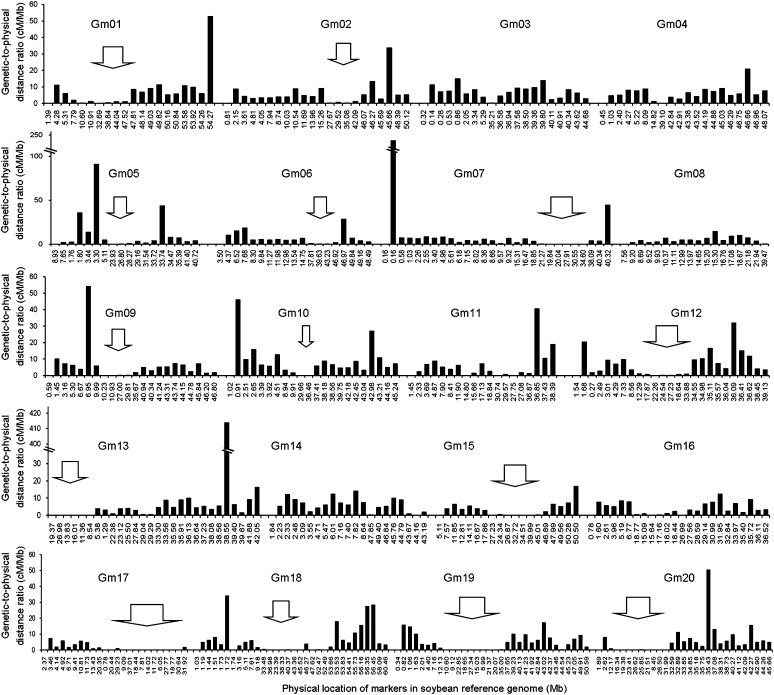
Distribution of genetic recombination frequencies along the chromosomes in soybean genome. In each plot, the horizontal axis (in Mb) represents the physical location along the reference chromosomes according to the order of markers in the present genetic map and the vertical axis (cM/Mb) represents the genetic-to-physical distance ratio. The regions with large suppression of recombination (genetic-to-physical distance ratio <2 cM/Mb and physical distance range >5 Mb) were marked by hollow arrows.

Investigating the association between genetic and physical distances of adjacent SNP markers revealed that the recombination frequency varied along the chromosome (Figure 4). The average genetic-to-physical distance ratio was 7.76 cM/Mb, with a range from 0 to 414.1 cM/Mb. Twenty hotspot regions with high frequencies of recombination (genetic-to-physical distance ratio >20 cM/Mb) were identified on the chromosomal arms of Gm01 (1), Gm02 (1), Gm04 (1), Gm05 (3), Gm06 (1), Gm07 (2), Gm09 (1), Gm10 (2), Gm11 (1), Gm12 (2), Gm13 (1), Gm18 (3), and Gm20 (1).

We observed low recombination frequencies (genetic-to-physical distance ratio <2 cM/Mb and physical distances >5 Mb) in 14 regions of the genome. All were located in the pericentromeric regions of chromosomes. In addition, 13 pairs of markers that were separated in the soybean reference genome (from 9.3 kb to 12.3 Mb with an average of 1.85 Mb of physical distance) had no observable recombination events between them. All pairs were located in pericentromeric regions, with 10 of them being included in recombination coldspots, with the exception of Map-2639 in Gm14, Map-3033 in Gm16, and satt505 in Gm18.

## Discussion

The traditional approach to select polymorphic PCR-based molecular markers in biparental populations is time-consuming. For instance, we tested 890 SSRs, mostly from the integrated linkage map of soybean (Song *et al.* 2004) for polymorphism between the parental inbred lines ZH and ZP. Only 19.2% of the SSRs, however, were polymorphic. To solve the issue of low polymorphism, we resequenced the genomes of ZH and ZP at 4.68-fold and 2.82-fold coverage respectively for SNP detection (Li *et al.* 2013). Using Glyma1.0 (Schmutz *et al.* 2010) as a reference sequence, 794,876 SNPs were identified across the genome. Consequently, resequencing closely related parents is a promising avenue to provide polymorphic markers for constructing high-density genetic map and fine-mapping QTL.

In addition, resequencing analysis provides us an opportunity to take a closer look at the landscape of polymorphisms among the two parental lines. Our analysis revealed that there are several regions with a low degree of polymorphisms between the two parents (Figure 1), which makes it difficult to screen for polymorphic markers in these areas. The potential miscalling of SNPs was reported previously to amount to 2.7% of misclassifications based on Sanger sequencing results (Li *et al.* 2013). Therefore, it is tempting to speculate that several of these large regions with a degree of polymorphisms below the misclassification rate might be identical by descent. This information is of particular relevance when using multiple-line crosses for QTL mapping, because it allows to estimate the maximum number of alleles segregating among parents (Jannink and Wu 2003).

Using SNPs developed from resequencing of parental lines offers the potential to develop customized SNP arrays optimized for the targeted germplasm. We tested this option and developed 384 stable SNPs from the 794,876 SNPs to genotype the 254 RILs available. High-quality data were generated for 81.5% of the 384 SNP loci, which is comparable to that of previous studies (Hyten *et al.* 2008; Li *et al.* 2010). The genetic linkage map encompassed 2594.34 cM, which is consistent with the third and fourth versions of the soybean integrated genetic linkage map as well as a high-density SSR integrated genetic linkage map (Hwang *et al.* 2009). The number of intervals void of markers >20 cM in the current map was lower as compared to that in the map based on 412 SSRs constructed for the “Minsoy” × “Noir 1” mapping population (Cregan *et al.* 1999). Consequently, this finding clearly underlines the potential to use resequencing data to design customized SNP arrays for QTL mapping.

Variation of recombination rates plays a key role in shaping genomic features and influencing the strength of natural selection (Gaut *et al.* 2007). The distribution of the genetic recombination frequency across the genome was analyzed based on the comparison of physical and genetic distances (Figure 4). We observed that the pericentromeric regions are coldspots of recombinations similar to that reported for rice (Chen *et al.* 2002), maize (Carels *et al.* 1995), wheat (Erayman *et al.* 2004), and soybean (Schmutz *et al.* 2010). The pericentromeric regions in soybean are gene-poor and recombinationally inactive, and most genes are clustered in the chromosome arms (Du *et al.* 2012). Nevertheless, the centromeric sequences within the pericentromeric regions are key factors for cell division and stable transmission of genetic information. Several active genes (Nagaki *et al.* 2004) and a series of QTL or genes related to important agronomy traits such as grain weight in rice have been identified in pericentromeric regions (Li *et al.* 2004). The severely suppressed recombination resulted in sharply reduced diversity in these regions and might have contributed to the formation of extended regions of sequence identity between the two parents. Despite the need to maintain conserved regions for stable cell division, widening the bottleneck of reduced numbers of recombinations in the centromeric regions might be an important aspect of breaking the current yield barriers in soybean breeding.

## Supplementary Material

Supporting Information
