# Supplementary material for: Biparental Resequencing Coupled With SNP Genotyping of a Segregating Population Offers Insights Into the Landscape of Recombination and Fixed Genomic Regions in Elite Soybean
Source: G3 (Bethesda). 2014 Jan 29;4(4):553–60. doi: 10.1534/g3.113.009589 (PMC4059229; doi:10.1534/g3.113.009589)
Supplement: Supporting Information [file supp_4_4_553__index.html]

Biparental Resequencing Coupled With SNP Genotyping of a Segregating Population Offers Insights Into the Landscape of Recombination and Fixed Genomic Regions in Elite Soybean — Supporting Information 

# Biparental Resequencing Coupled With SNP Genotyping of a Segregating Population Offers Insights Into the Landscape of Recombination and Fixed Genomic Regions in Elite Soybean

## Supporting Information for Li *et al.*, 2014

**Files in this Data Supplement:**

- Table S1 - Information underlying the current genetic linkage map including 484 molecular markers (.xlsx, 109 KB)
